# *Schistosoma japonicum* peptide SJMHE1 inhibits acute and chronic colitis induced by dextran sulfate sodium in mice

**DOI:** 10.1186/s13071-021-04977-y

**Published:** 2021-09-06

**Authors:** Wenqi Shan, Wenzhe Zhang, Fei Xue, Yongbin Ma, Liyang Dong, Ting Wang, Yu Zheng, Dingqi Feng, Ming Chang, Guoyue Yuan, Xuefeng Wang

**Affiliations:** 1 Department of Central Laboratory, The Affiliated Hospital of Jiangsu University, Zhenjiang, Jiangsu China; 2 Department of Pediatrics, The Affiliated Hospital of Jiangsu University, Zhenjiang, Jiangsu China; 3 Department of Blood Transfusion, The Affiliated Hospital of Jining Medical University, Jining, Shandong China; 4 Department of Central Laboratory, Jintan Hospital, Jiangsu University, Jintan, Jiangsu China; 5 Department of Nuclear Medicine and Institute of Oncology, The Affiliated Hospital of Jiangsu University, Zhenjiang, Jiangsu China; 6 Department of Endocrinology, Affiliated Hospital of Jiangsu University, Zhenjiang, Jiangsu China

**Keywords:** *Schistosoma japonicum* peptide, SJMHE1, Inhibit, Acute and chronic colitis

## Abstract

**Background:**

Harnessing helminth-based immunoregulation is a novel therapeutic strategy for many immune dysfunction disorders, including inflammatory bowel diseases (IBDs). We previously identified a small molecule peptide from *Schistosoma japonicum* and named it SJMHE1. SJMHE1 can suppress delayed-type hypersensitivity, collagen-induced arthritis and asthma in mice. In this study, we assessed the effects of SJMHE1 on dextran sulfate sodium (DSS)-induced acute and chronic colitis.

**Methods:**

Acute and chronic colitis were induced in C57BL/6 mice by DSS, following which the mice were injected with an emulsifier SJMHE1 or phosphate-buffered saline. The mice were then examined for body weight loss, disease activity index, colon length, histopathological changes, cytokine expression and helper T (Th) cell subset distribution.

**Results:**

SJMHE1 treatment significantly suppressed DSS-induced acute and chronic colitis, improved disease activity and pathological damage to the colon and modulated the expression of pro-inflammatory and anti-inflammatory cytokines in splenocytes and the colon. In addition, SJMHE1 treatment reduced the percentage of Th1 and Th17 cells and increased the percentage of Th2 and regulatory T (Treg) cells in the splenocytes and mesenteric lymph nodes of mice with acute colitis. Similarly, SJMHE1 treatment upregulated the expression of interleukin-10 (IL-10) mRNA, downregulated the expression of IL-17 mRNA and modulated the Th cell balance in mice with chronic colitis.

**Conclusions:**

Our data show that SJMHE1 provided protection against acute and chronic colitis by restoring the immune balance. As a small molecule, SJMHE1 might be a novel agent for the treatment of IBDs without immunogenicity concerns.

**Graphical abstract:**

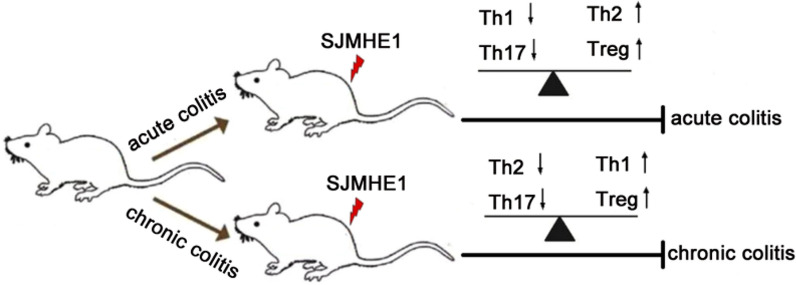

## Background

Inflammatory bowel diseases (IBDs), including ulcerative colitis (UC) and Crohn’s disease (CD), are chronic and inflammatory diseases for which there is no apparent cause and no real cure. In recent years the incidence of IBD has steadily increased worldwide, including in China [1]. Patients with these conditions are mainly treated with conventional medications, including corticosteroids (e.g. budesonide and prednisone) and immunomodulators (e.g. azathioprine and methotrexate). Cytokines, monoclonal antibodies, cytokine antagonists and soluble receptors are used to treat patients with IBD and those with poor or no response to conventional drugs [2, 3]. However, almost all available drugs for the treatment of IBD have adverse side effects. Biologics, such as cytokines and antibodies, have high manufacturing costs that represent a major challenge to their use in IBD management [1, 4, 5]. Thus, novel therapeutics for IBD that are safe and have minimal costs should be developed.

The hygiene hypothesis has been confirmed to provide possible factors that are associated with the increasing incidence of IBD. Studies have shown that exposure to infections has been reduced in recent years, especially exposure to helminth infection, as a result of improved sanitation, vaccination and the use of antibiotics. One suggestion is that the rapid increase in the incidence of IBD may be correlated with the reduced incidence of helminth infection [6, 7]. Helminths have co-evolved with human hosts and developed various mechanisms to modulate the immune network of their hosts [8]. Helminth-driven immunoregulation has been harnessed to treat patients with IBD [9]. *Schistosoma mansoni* infection fails to induce colitis in mice when trinitrobenzenesulfonic acid (TNBS) is administered through the intrarectal route [10]. In addition, TNBS-induced colitis in mice can be decreased by the oral administration of *Schistosoma japonicum* (*S. japonicum*) eggs [11]. In one study, 29 patients with active CD were administered *Trichuris suis* ova (TSO) at 3-weeks intervals for 24 weeks. On week 24, the Disease Activity Index (DAI) of CD in 23 patients had decreased, and 21 of the 29 patients were in remission. Furthermore, no adverse events occurred [12]. Helminths also secrete immunomodulatory molecules, including glycans, proteins, lipids and nucleic acids, that have been confirmed to protect mice against colitis. Excretory/secretory (ES) products from the hookworm *Ancyclostoma caninum* can suppress TNBS-induced colitis in mice by inhibiting inflammatory cytokine production [13]. SJMHE1, an anti-inflammatory peptide from the HSP60 protein of *S. japonicum*, suppresses delayed-type hypersensitivity [14], collagen-induced arthritis [15] and asthma [16] in mice. However, the use of live helminths or helminth ES products carries the risk for potential infection or immunogenicity, respectively. Thus, a small molecule peptide from helminths, such as SJMHE1, should be safer and less expensive than biologics for the treatment of IBD and has the potential to be the next-generation drugs.

In the present study, we used the dextran sulphate sodium (DSS) model of colitis to demonstrate that SJMHE1 provided protection against inducible acute and chronic colitis in mice. We found that SJMHE1 treatment regulated cytokine expression in the colon and splenocytes and also modulated helper T (Th) cell balance in splenocytes and mesenteric lymph nodes (MLNs) in mice with acute and chronic colitis. These results suggest that SJMHE1 may be considered to be a small molecule peptide candidate for a novel treatment for IBD.

## Methods

### Mice

C57BL/6 male mice aged 6–8 weeks (each weighing 19–22 g) were purchased from Cavins Experimental Animals Co., Ltd. (Changzhou, China; No. 201911131). They were fed at a specific pathogen-free level in the Experimental Animal Center at Jiangsu University. Animal experiments were conducted in accordance with the Guidelines for the Care and Use of Laboratory Animals and approved by the Animal Research Ethics Committee of Jiangsu University (UJS-LEAR-AP-2018030601).

### Peptides

*Schistosoma japonicum* peptide SJMHE1 was synthesized and purified by ChinaPeptides (Shanghai, China) as described previously [14–16]. Once synthesized, SJMHE1 was pretreated with polymyxin B-agarose to remove possible lipopolysaccharide contamination, as previously described [14–16]. The peptide was detected using high-performance liquid chromatography with a purity of > 98% and stored at − 80 °C until use.

### Experimental colitis induction with DSS and SJMHE1 treatment

Male C57BL/6 mice were randomly divided into four groups: normal (control), DSS, DSS/PBS and DSS/SJMHE1 groups. Both acute and chronic colitis were induced in the mice by adding DSS (molecular weight: 36–50 kDa; MP Biomedicals, Santa Ana, CA, USA) to the drinking water. To induce acute colitis, the mice were provided with drinking water containing 2.5% DSS continuously for 10 days, in accordance with a previously described method [17]. The mice in the DSS/SJMHE1 and DSS/PBS groups were subcutaneously injected with 0.1 ml emulsified SJMHE1 (10 µg) or PBS with incomplete Freund’s adjuvant (IFA; Sigma, Poole, UK), respectively, on days 0 and 7. To induce chronic colitis, the mice were provided with drinking water containing 2% DSS on days 0–5, 10–15 and 20–25, as described previously [17]. In the DSS/SJMHE1 and DSS/PBS groups, the mice were treated with 0.1 ml emulsified SJMHE1 (10 µg) or PBS with IFA, respectively, on days 0, 14 and 28. During treatment, the mice were monitored daily for fecal traits, blood in stool and weight change. The DAI was calculated in accordance with the scoring criteria of K Yoshihara and colleague, as described in [17]. The mice were then anesthetized and killed to evaluate the length and inflammation of the colon.

### Histopathological analysis

Colon tissue segments (approx. 0.5 cm) were collected and fixed in a formalin solution at room temperature for 2 days. These segments were then dehydrated, embedded in paraffin, sectioned and stained with hematoxylin and eosin (H&E). The pathological changes and inflammation of colon tissues were assessed using a standard histopathological score, as previously described [18].

### Flow cytometry analysis

The spleen and MLNs were aseptically separated, and single-cell suspensions were prepared as described previously [14–16]. Single-cell suspensions from the spleen and MLNs were stimulated with a brefeldin/monensin mixture [Brefeldin A; Multisciences (Lianke) Biotech, Hangzhou, China] and a phorbol-12-myristate-13-acetate/ionomycin mixture [Multisciences (Lianke) Biotech] for 5 h. Fluorescein isothiocyanate (FITC)-anti-mouse-CD4 (eBioscience, Thermo Fisher Scientific, Waltham, MA, USA) was used for surface staining. APC-anti-mouse-IFN-γ (eBioscience, Thermo Fisher Scientific), APC-anti-mouse-IL-4 (BioLegend, San Diego, CA, USA) and PE-anti-mouse-IL-17A (Biolegend) were utilized for intracellular staining after the membrane was ruptured by fixation/permeabilization (BD Biosciences, Franklin Lakes, NJ, USA) to analyze Th1, Th2 and Th17 cells as described previously [16, 19]. Mononuclear cells were stained with FITC-anti-mouse-CD4, APC-anti-mouse-CD25 and PE-anti-mouse Foxp3 by using a mouse regulatory T cell staining kit (eBioscience) to evaluate regulatory T (Treg) cells. All samples were analyzed using a BD FACSCcanto flow cytometer (BD Biosciences), and data were analyzed with Flowjo v10.0.7 software (Tree Star, Ashland, OR, USA).

### RNA extraction and quantitative reverse transcription-PCR

Total RNA was extracted from the spleen cells by using a TRizol reagent (Invitrogen, Fisher Price Scientific, Waltham, MA, USA). First-strand cDNA was synthesized with a reverse transcription kit (GeneCopoeia, Germantown, MD, USA). RNA was isolated from the colon tissue by using a tissue RNA purification kit Plus (Yishan Biological Technology, Shanghai, China). The cDNA of the mRNA was synthesized with a reverse transcription kit (TakaRa, Tokyo, Japan). All-in-one™ qPCR primer sets for interferon gamma (IFN-γ; Cat. No. MQP027401), interleukin-4 (IL-4; Cat. No. MQP032451), IL-17A (Cat. No. MQP029457), IL-10 (Cat. No. MQP029453), transforming growth factor beta (TGF-β; Cat. No. MQP030343), IL-35 (Cat. No. MQP027412) and glyceraldehyde 3-phosphate dehydrogenase (GAPDH; Cat. No. MQP027158) were obtained from GeneCopoeia Inc. (Rockville, Md, USA). The PCR amplification and calculation of the relative mRNA expression were based on previously described methods [16, 19].

### Statistical analysis

Data were expressed as the mean ± standard error of the mean, and statistical significance was analyzed through one-way analysis of variance followed by Dunn’s multiple comparison with GraphPad Prism 8.0.0 (GraphPad Software Inc., San Diego, CA, USA). Data with *P* < 0.05 were considered to be statistically significant.

## Results

### SJMHE1 treatment alleviates DSS-induced acute colitis in mice

**Fig. 1:**
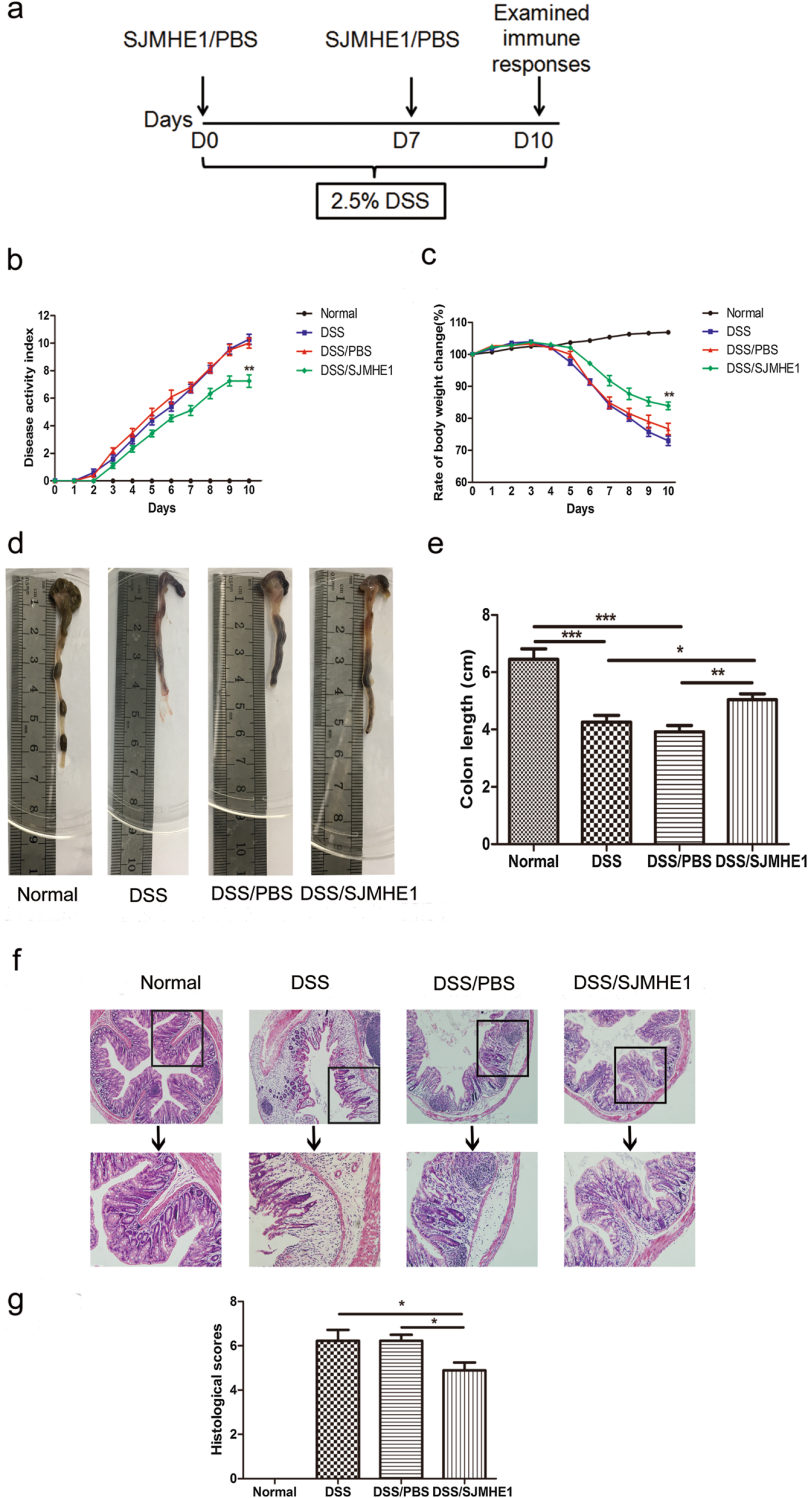
Treatment with the *Schistosoma japonicum* small molecule peptide, SJMHE1, alleviated colon inflammation in mice with DDS-induced acute colitis. **a** Experimental scheme. C57BL/6 mice were provided with drinking water containing 2.5% DSS for 10 days. They were injected with SJMHE1 or PBS emulsified in IFA on days 0 and 7. The mice were killed on day 10. **b**Disease activity index (**b**) and rate of body weight change (**c**) in each group. **d** Macroscopic appearance of colon. **e** Colon length in each group. **f** Histological analysis of the colon from mice with H&E staining (magnification 20×). Images represent the results of three independent experiments (total *n* = 18 mice, 6 mice per group). **g** Histological scores were assessed from each mouse. Data are presented as mean ± standard error of the mean (SEM; *n* = 18) of three independent experiments. Asterisks indicate significant difference at: *P < 0.05, **P < 0.01, ***P < 0.001

Acute colitis was induced in mice provided with 2.5% DSS in the drinking water continuously for 10 days. The treatment regimen is illustrated in Fig. 1a. Treatment with SJMHE1 alleviated colitis in mice, as indicated by a decrease in the DAI, decreased weight loss and decreased shortening of the colon (Fig. 1b–e). Histological analysis of the colons of these mice showed that SJMHE1 treatment significantly reduced the infiltration of inflammatory cells, mucosal injury and edema (Fig. 1f). The histological score of the colons was lower in SJMHE1-treated mice than in the DSS and DSS/PBS groups (Fig. 1g). These results indicated that SJMHE1 treatment attenuated the disease activity in mice with DSS-induced acute colitis.

### SJMHE1 treatment regulates cytokine expression in the splenocytes and colon in DSS-induced acute colitis in mice

**Fig. 2:**
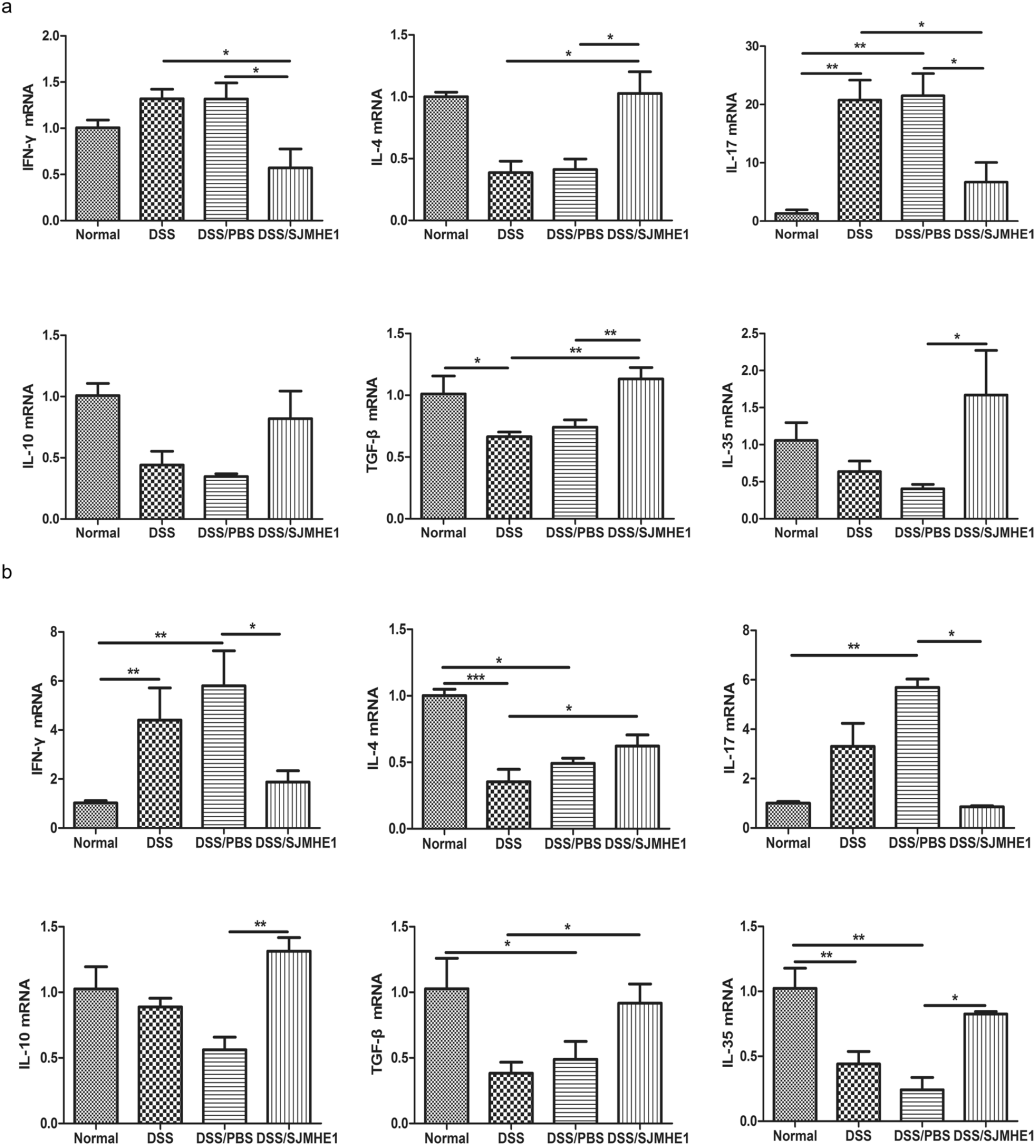
SJMHE1 treatment regulates the expression of cytokines from the splenocytes and colon of mice with DSS-induced acute colitis. On day 10, the mice were killed, and the splenocytes (**a**) and colon (**b**) from each mouse were tested for mRNA expression levels of IFN-γ, IL-4, IL-17, IL-10, TGF-β and IL-35 by reverse transcription quantitative PCR (qRT-PCR). Data are presented as the mean ± SEM of 18 mice from three independent experiments. Asterisks indicate significant difference at: *P < 0.05, **P < 0.01, ***P < 0.001. *IFN *Interferon,* IL* interleukin,* TGF* transforming growth factor

Pro-inflammatory cytokines play a key role in the pathogenesis of IBD [20, 21]; for example, helminth infection can evoke the production of regulatory cytokines to protect against inflammatory diseases, including IBD [7, 9, 22]. Thus, we investigated the expression of cytokines in the splenocytes and colon of mice from the normal (control), DSS, DSS/PBS and DSS/SJMHE1 groups. The expression level of IL-17 mRNA was higher in the splenocytes of the DSS and DSS/PBS groups than in those of the normal (control) group, and the expression level of TGF-β mRNA was lower in the splenocytes of the DSS group than in those of the normal (control) group (Fig. 2a). However, the expression levels of IL-4 and TGF-β mRNA in the splenocytes of the DSS/SJMHE1 group were upregulated and those of interferon gamma (IFN-γ) and IL-17 mRNA were downregulated compared with those in the DSS and DSS/PBS groups. The expression level of IL-35 mRNA was higher in the DSS/SJMHE1 group than in the DSS/PBS group (Fig. 2a). The expression level of IFN-γ mRNA in the colon of the DSS and DSS/PBS groups was higher than that in the normal (control) group. The expression levels of IL-4 and IL-35 mRNA were lower in the DSS and DSS/PBS groups than in the normal (control) group (Fig. 2b). The expression of IL-17 mRNA in the DSS/PBS was increased, whereas the expression of TGF-β mRNA was decreased compared to the normal (control) group (Fig. 2b). However, the expression levels of IFN-γ and IL-17 mRNA were lower in the colons of mice in the DSS/SJMHE1 group than in those of mice the DSS/PBS group. The expression levels of IL-4, IL-10, TGF-β and IL-35 mRNA in the DSS/SJMHE1 group were higher than those in the DSS or DSS/PBS group (Fig. 2b). These results suggest that SJMHE1 treatment could regulate the expression of cytokines in the splenocytes and colons of mice with acute colitis.

### SJMHE1 treatment modulates Th cell balance in the splenocytes and MLNs in mice with DDS-induced acute colitis

**Fig. 3:**
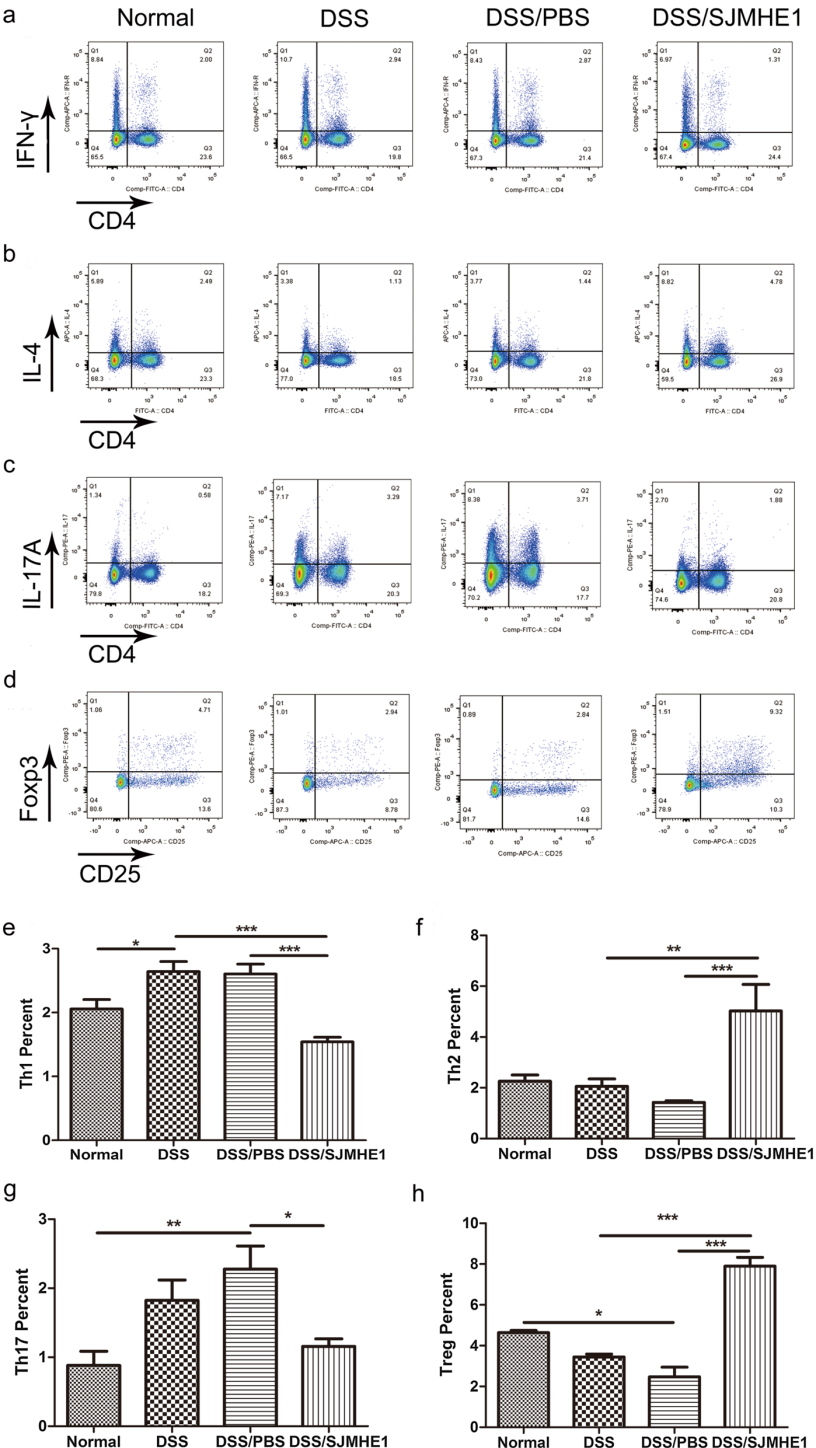
SJMHE1 treatment regulates Th1/Th2/Th17/Treg cell distribution from the splenocytes of mice with DSS-induced acute colitis. On day 10, the mice were killed, and splenocytes from each mouse were tested for Th1/Th2/Th17/Treg subsets through flow cytometry.** a**–**d** Proportion of Th1/Th2/Th17/Treg subsets in each treatment group: **a** CD4^+^IFN-γ^+^ Th1 cells, **b** CD4^+^IL-4^+^ Th2 cells, **c** CD4^+^IL-17^+^ Th17 cells, **d** CD4^+^CD25^+^Foxp3^+^ Treg cells. Data are representative of the results of the experiments.** e**–**h** Percentages of Th1/Th2/Th17/Treg cells in each treatment group: **e** CD4^+^IFN-γ^+^ Th1 cells, **f** CD4^+^IL-4^+^ Th2 cells, **g** CD4^+^IL-17^+^ Th17 cells, **h** CD4^+^CD25^+^Foxp3^+^ Treg cells. Results are presented as the mean ± SEM of 18 mice from three independent experiments. Asterisks indicate significant difference at: *P < 0.05, **P < 0.01, ***P < 0.001

**Fig. 4:**
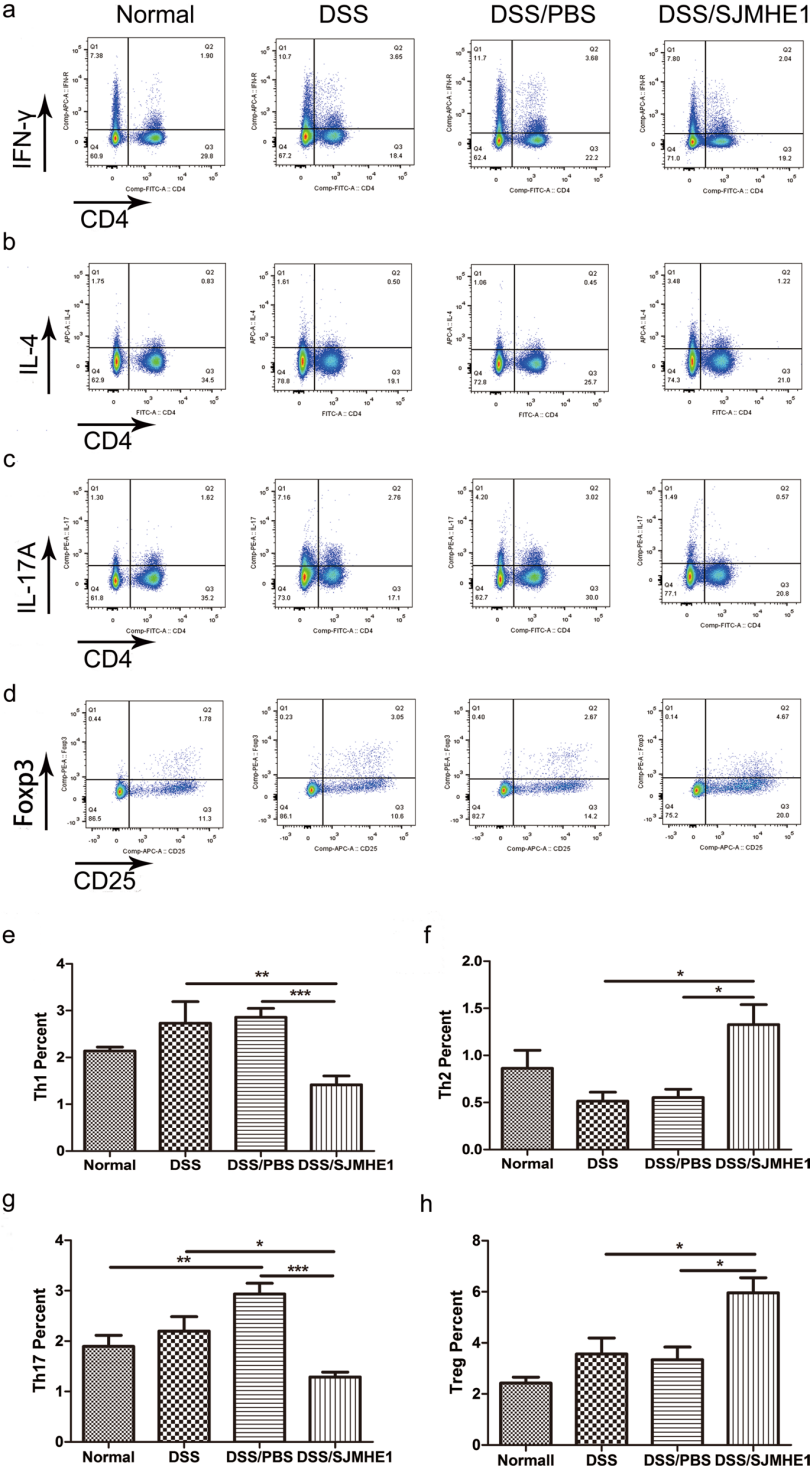
SJMHE1 treatment modulates Th1/Th2/Th17/Treg cell distribution in the MLNs of mice with DSS-induced acute colitis. On day 10, the mice were killed, and the MLNs from each mouse were tested for Th1/Th2/Th17/Treg subsets through flow cytometry.** a**–**d** Proportion of Th1/Th2/Th17/Treg subsets in each treatment group: **a** CD4^+^IFN-γ^+^ Th1 cells, **b** CD4^+^IL-4^+^ Th2 cells, **c** CD4^+^IL-17^+^ Th17 cells, **d** CD4^+^CD25^+^Foxp3^+^ Treg cells. Data are representative of the experiments.** e**–**h** Percentages of Th1/Th2/Th17/Treg cells in each treatment group: **e** CD4^+^IFN-γ^+^ Th1 cells, **f** CD4^+^IL-4^+^ Th2 cells, **g** CD4^+^IL-17^+^ Th17 cells, **h** CD4^+^CD25^+^Foxp3^+^ Treg cells. Results are presented as mean ± SEM of 18 mice from three independent experiments. Asterisks indicate significant difference at: *P < 0.05, **P < 0.01, ***P < 0.001

Dysregulated CD4^+^ T cells have also been demonstrated to play a crucial role in the pathogenesis of IBD [23, 24]. The Th subsets were detected in the splenocytes and MLNs of the mice from the normal (control), DSS, DSS/PBS and DSS/SJMHE1 groups. As shown in Fig. 3, the proportions of CD4^+^IFN-γ^+^ Th1 and CD4^+^IL-17^+^ Th17 cells increased in the splenocytes of the DSS groups compared with the normal (control) group. However, SJMHE1 treatment significantly reduced the proportions of Th1 and Th17 cells and increased the percentage of CD4^+^IL-4^+^ Th2 and CD4^+^CD25^+^Foxp3^+^ Treg cells in the splenocytes compared with those in the DSS and/or DSS/PBS groups (Fig. 3). The proportions of Th1 and Th17 cells decreased and the proportions of Th2 and Treg cells increased in the MLNs of the mice in the DSS/SJMHE1 group (Fig. 4). These results indicate that SJMHE1 treatment downregulated Th1 and Th17 cells and upregulated Th2 and Treg cells, suggesting that SJMHE1 treatment might provide protection to mice against DSS-induced acute colitis.

### Improvement of DSS-induced chronic colitis in SJMHE1-treated mice

**Fig. 5:**
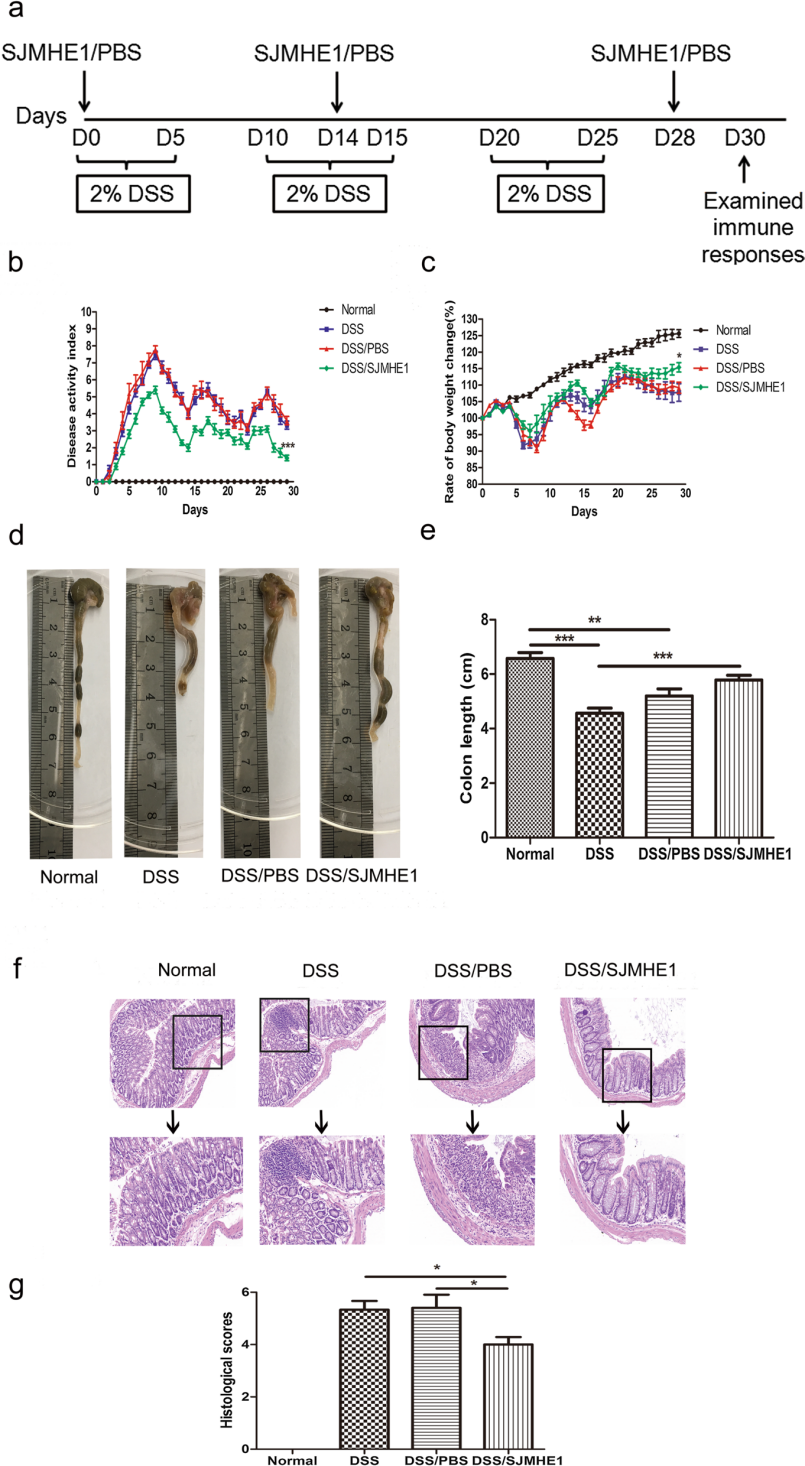
SJMHE1 treatment protects against the induction of chronic colitis by DSS in mice. **a** Experimental scheme. C57BL/6 mice were provided with drinking water containing 2% DSS on days 0–5, 10–15, and 20–25. Mice were injected with SJMHE1 or PBS emulsified in IFA on days 0, 14 and 28. The mice were killed on day 30.** b** DAI (**b**) and rate of body weight change (**c**) in each group. **d** Macroscopic appearance of the colon. **e** Colon length in each group. **f** Histological analysis of the colon from mice by H&E staining (magnification 20×). Images represent three independent experiments (total *n* = 18 mice, 6 mice per group. **g** Histological scores were assessed from each mouse. Data are presented as the mean ± SEM (*n* = 18) from three independent experiments. Asterisks indicate significant difference at: *P < 0.05, **P < 0.01, ***P < 0.001

To detect the effects of SJMHE1 treatment on chronic colitis, we first induced chronic colitis in the mice by adding 2% DSS to the drinking water on days 0–5, 10–15, and 20–25 [17]. The treatment regimen is shown in Fig. 5a. Similar to the effect of SJMHE1 on acute colitis, SJMHEI treatment was followed by a decrease in the DAI, decreased weight loss and decreased shortening of the colon (Fig. 5b–e). The crypt damage, ulceration and infiltration of inflammatory cells present in DSS- and DSS/PBS-treated mice were detected by histological analysis. SJMHE1 treatment decreased the degree of mucosal injury, the number of infiltrating cells and the histological scores compared with those in the DSS and DSS/PBS groups (Fig. 5f, g). These results suggest that SJMHE1 could suppress DSS-induced chronic colitis in mice.

### SJMHE1 treatment decreased the expression of IL-17 mRNA and increased the expression of IL-10 mRNA in the splenocytes and colon of mice with DSS-induced chronic colitis

**Fig. 6:**
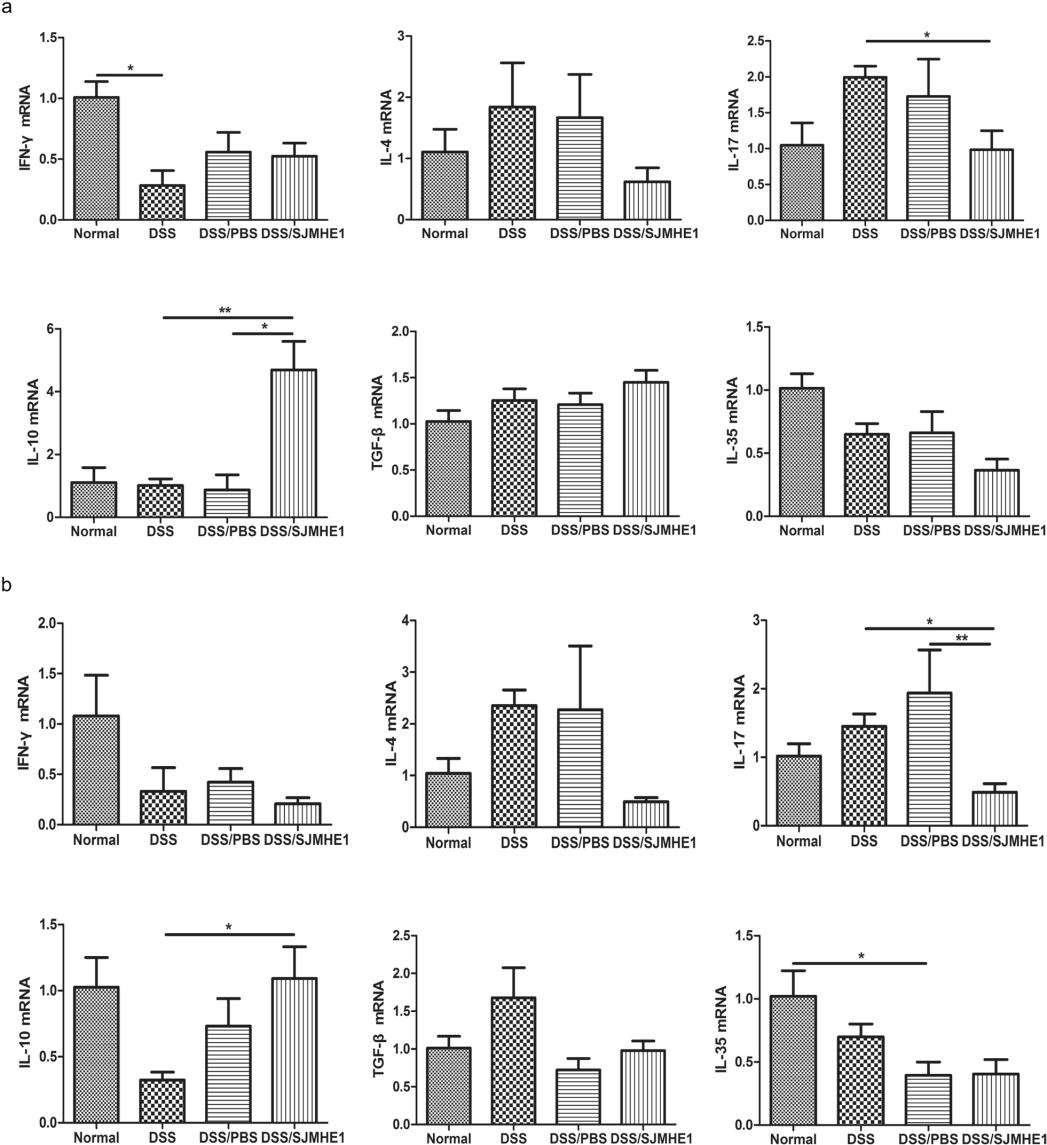
SJMHE1 treatment regulates the expression of cytokines from the splenocytes and colons of mice with DSS-induced chronic colitis. On day 30, the mice were killed, and the splenocytes (**a**) and colon (**b**) from each mouse were tested for the expression levels of IFN-γ, IL-4, IL-17, IL-10, TGF-β and IL-35 mRNA by qRT-PCR. Data are presented as mean ± SEM of 18 mice from three independent experiments. Asterisks indicate significant difference at: *P < 0.05, **P < 0.01, ***P < 0.001

The expression of cytokines in the splenocytes and colon was examined to evaluate the cytokine production during SJMHE1 treatment in mice with DSS-induced chronic colitis. As shown in Fig. 6, the expression level of IFN-γ mRNA in the splenocytes of the DSS group was less than that in the splenocytes of the normal (control) group. Compared with the normal (control) group, the mRNA expression level of IL-17 in the DSS group was slightly increased but the difference was not statistically significant. SJMHE1 treatment reduced the expression level of IL-17 mRNA and increased that of IL-10 mRNA compared with their levels in the DSS and/or DSS/PBS group. The IL-4 level slightly increased in the DSS and DSS/PBS groups compared with the normal (control). SJMHE1 treatment reduced the expression of IL-4 mRNA, but this decrease was not statistically significant (Fig. 6a). Similarly, SJMHE1 treatment downregulated the expression of IL-17 mRNA and upregulated that of IL-10 mRNA in the colon of mice compared with the respective levels in the DSS/PBS or DSS group (Fig. 6b). These results suggest that the inhibitory effect of SJMHE1 on DSS-induced chronic colitis might be associated with the modulation of the expression levels of IL-17 and IL-10 mRNA.

### SJMHE1 treatment regulated the distribution of Th cells in the splenocytes and MLNs of mice with DSS-induced chronic colitis

**Fig. 7:**
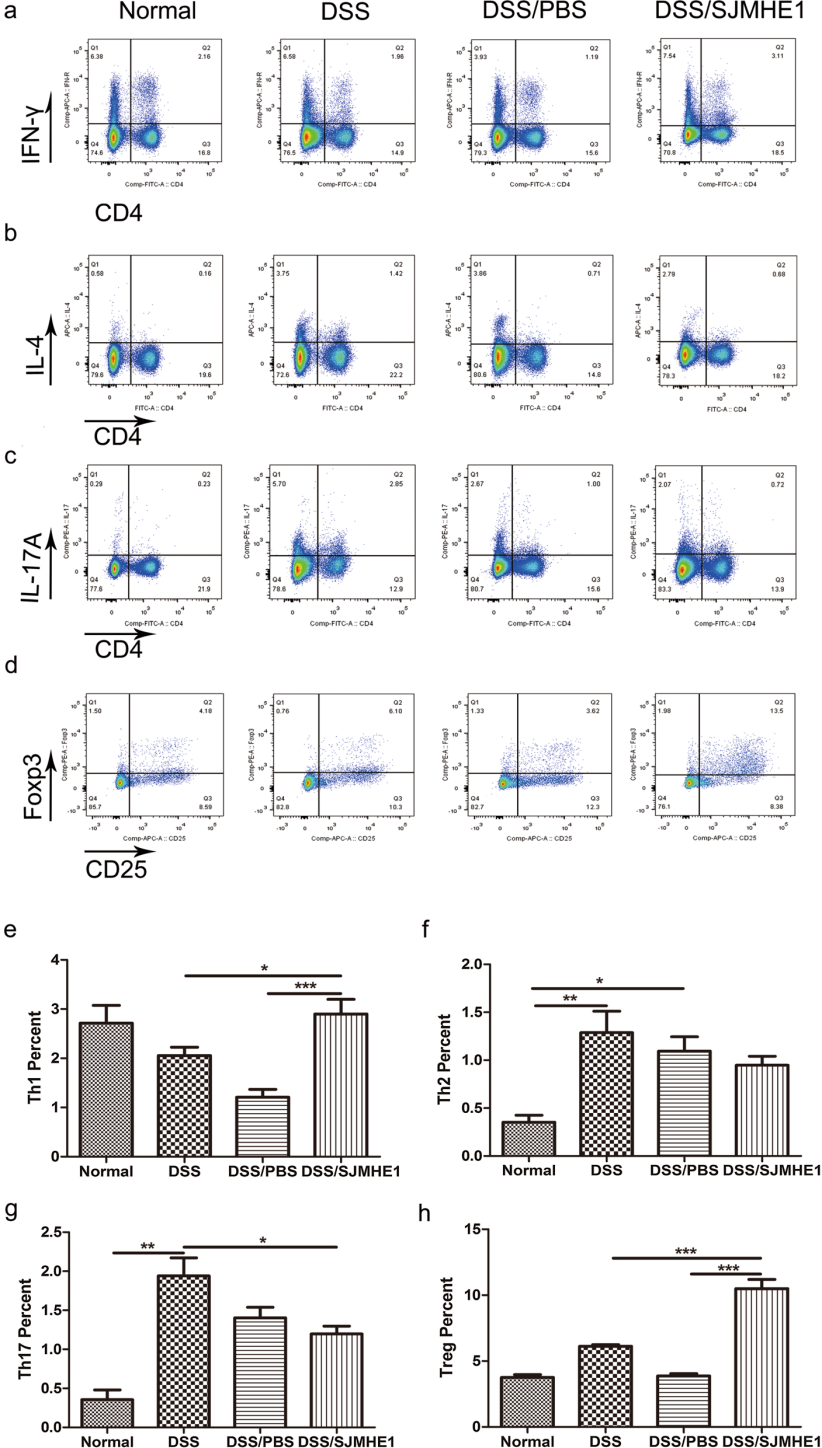
SJMHE1 treatment regulates Th1/Th2/Th17/Treg cell distribution from the splenocytes of mice with DSS-induced chronic colitis. On day 30, the mice were killed, and splenocytes from each mouse were tested for Th1/Th2/Th17/Treg subsets through flow cytometry.** a**–**d** Proportion of Th1/Th2/Th17/Treg subsets in each group: **a** CD4^+^IFN-γ^+^ Th1 cells, **b** CD4^+^IL-4^+^ Th2 cells, **c** CD4^+^IL-17^+^ Th17 cells, **d** CD4^+^CD25^+^Foxp3^+^ Treg cells. Data are representative of the results of the experiments.** e**–**h** Percentages of Th1/Th2/Th17/Treg cells in each treatment group: **e** CD4^+^IFN-γ^+^ Th1 cells, **f** CD4^+^IL-4^+^ Th2 cells, **g** CD4^+^IL-17^+^ Th17 cells, **h** CD4^+^CD25^+^Foxp3^+^ Treg cells. Results are presented as mean ± SEM of 18 mice from three independent experiments. Asterisks indicate significant difference at: *P < 0.05, **P < 0.01, ***P < 0.001

**Fig. 8:**
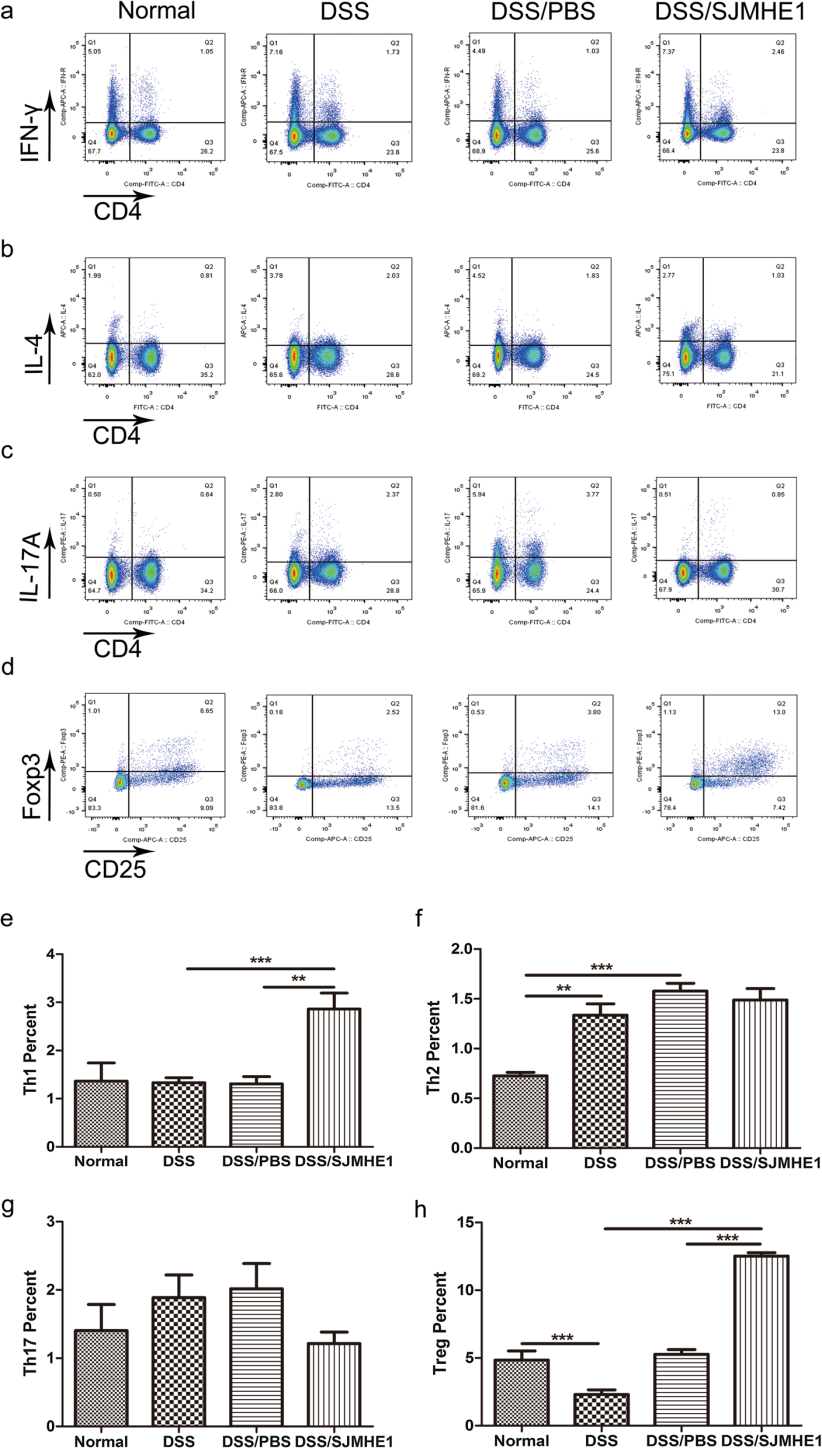
SJMHE1 treatment regulates Th1/Th2/Th17/Treg cell distribution from the MLNs of mice with DSS-induced chronic colitis. On day 30, the mice were killed, and splenocytes from each mouse were tested for Th1/Th2/Th17/Treg subsets through flow cytometry.** a**–**d** Proportion of Th1/Th2/Th17/Treg subsets in each group: **a** CD4^+^IFN-γ^+^ Th1 cells, **b** CD4^+^IL-4^+^ Th2 cells, **c** CD4^+^IL-17^+^ Th17 cells, and **d** CD4^+^CD25^+^Foxp3^+^ Treg cells. Data are representative of the experiments.** e**–**h** Percentages of Th1/Th2/Th17/Treg cells in each treatment group: **e** CD4^+^IFN-γ^+^ Th1 cells, **f** CD4^+^IL-4^+^ Th2 cells, **g** CD4^+^IL-17^+^ Th17 cells, **h** CD4^+^CD25^+^Foxp3^+^ Treg cells. Results are presented as the mean ± SEM of 18 mice from three independent experiments. Asterisks indicate significant difference at: *P < 0.05, **P < 0.01, ***P < 0.001

The Th cells in the splenocytes and MLNs were examined by flow cytometry to test the effect of SJMHE1 treatment on the Th subsets of mice with DSS-induced chronic colitis. As shown in Fig. 7, the percentages of CD4^+^IL-4^+^ Th2 and CD4^+^IL-17^+^ Th17 cells in the splenocytes of the DSS-treated mice increased compared with those in normal (control) mice; however, SJMHE1 treatment significantly reduced the proportions of Th17 cells and increased the proportions of Th1 and Treg cells in the splenocytes compared with those in the DSS group. Consistent with the decrease in the expression of IL-4 mRNA (Fig. 6b), decreased proportions of Th2 cells were induced by SJMHE1 treatment in the splenocytes, but this decrease was not significant. Furthermore, the DSS-treated mice were found to have increased percentages of Th2 cells and decreased percentages of Treg cells in their MLNs compared with the normal (control) mice (Fig. 8). SJMHE1 treatment also increased the percentage of Th1 and Treg cells in MLNs compared with that of the DSS and DSS/PBS groups. However, SJMHE1 treatment slightly decreased the proportion of Th17 cells in MLNs compared with that in the DSS or DSS/PBS group (Fig. 8). These results suggest that SJMHE1 suppressed DSS-induced chronic colitis partly mediated by regulating the balance of Th cells.

## Discussion

Inflammatory bowel diseases, including CD and UC, are characterized by chronic relapsing inflammation with intestinal epithelial injury and immune homeostasis disruption. The pathogenesis of IBD has not yet been identified, and available treatments for IBD remain limited. Helminth therapy for IBD has been shown to inhibit colitis in different animal models. The pig whipworm TSO has been assessed in patients with CD and UC in phase 1 trials. TSO significantly improved the symptoms of colitis in patients compared with those who received placebo after 12 weeks of therapy [25]. In another study, patients with CD and percutaneous infection of low doses of *Necator americanus* (*N. americanus*), their DAI decreased at week 20, and were in remission at week 45 after first inoculation with *N. americanus*. [26]. Further research found that ES products from helminths also have therapeutic properties. Soluble proteins from *S. mansoni* and *A. caninum* can ameliorate TNBS-induced colitis in mice [13]. Crude and ES products from *Ancylostoma ceylanicum* can also inhibit DSS-induced colitis in mice [27]. Although ES products instead of helminth infection can avoid many drawbacks of currently available helminth therapies, crude helminth products as a drug are limited [8]. A recombinant of anti-inflammatory protein from *A*. *caninum* (AIP-1) can suppress TNBS-induced colitis in mice [28]. An increasing number of immunomodulatory proteins from helminths have been identified, including ES-62 from *Acanthocheilonema vitae* that contains phosphorylcholine (PC), the bioactive moiety of ES-62. Small drug-like analogs of PC have therapeutic potential for arthritis and lung fibrosis, but they can avoid the immunogenicity concerns associated with treatment the whole ES-62 protein [29, 30]. Thus, helminth-derived anti-inflammatory small molecules for the treatment of colitis should be further explored.

In this study, SJMHE1, a small-molecule peptide from *S. japonicum*, was used to treat mice with DSS-induced acute and chronic colitis. The results revealed that SJMHE1 treatment significantly reduced the DAI and weight loss and shortening of the colon of the mice with acute and chronic colitis. This treatment also improved the survival rate of the mice with acute colitis (data not shown). The mice with colitis showed cellular infiltration, epithelial erosion and villus atrophy in colon tissues, but SJMHE1 treatment significantly protected the mice against DSS-induced gut pathology. Thus, SJMHE1 alleviated disease activity and inflammatory response in these mice with acute colitis induced by DSS.

Many pro-inflammatory cytokines play a crucial role in the pathogenesis of IBD. The pathogenesis of CD and UC is usually considered to be different, with CD is dominated by IFN-γ and IL-17A from Th1 and Th17 cells, respectively, and UC mediated by IL-4 and IL-13 from Th2 cells [1]. Consistent with the findings in mice with DSS-induced acute colitis [31–33], the expression of IL-17 mRNA in the splenocytes and of IFN-γ and IL-17 mRNA in the colon increased in the DSS and/or DSS/PBS groups. However, SJMHE1 treatment reduced the expression levels of IFN-γ and IL-17 mRNA, increased the expression levels of IL-4, TGF-β and IL-35 mRNA in the splenocytes and increased the expression levels of IL-4, IL-10, TGF-β and IL-35 mRNA in the colon of mice. SJMHE1 administration provided protection against DSS-induced acute colitis in mice. This observation might be related to the regulation of cytokine expression by SJMHE1. Similar results have been reported for soluble egg antigens (SEA) from* S. mansoni* and cystatin and Sj16 from *S. japonicum* [33–35].

Dysregulated CD4^+^ T cells have also been reported to play key role in the pathogenesis of IBD. Th1 and Th17 responses dominate in patients with CD, whereas Th2 response is mediated in patients with UC [1]. DSS-induced colitis was originally considered to be a T cell-independent model [36]. However, subsequent studies suggested that DSS colitis is a Th1- or Th17-mediated inflammation [37, 38]. Consistent with these studies, the acute model of colitis in our study demonstrated that the proportions of Th1 and Th17 cells in the splenocytes of the DSS and DSS/PBS groups were higher than those in the normal (control) mice (Fig. 3). The percentage of Th17 cells in the MLNs of the DSS/PBS group was higher than that in the normal (control) mice (Fig. 4). However, SJMHE1 treatment reduced the proportions of Th1 and Th17 cells and increased the percentage of Th2 and Treg cells in the splenocytes and MLNs (Fig. 4). Helminth infection or their products can skew the immune response toward Th2 and Treg responses, which are generally considered to suppress Th1- and Th17-mediated inflammation, including IBD [7, 39]. As a small molecule peptide from helminths, SJMHE1 inhibits the development of DSS-induced acute colitis through the induction of a Th2/Treg profile.

In the present study, SJMHE1 had a protective effect on DSS-induced chronic colitis in mice. SJMHE1 administration attenuated clinical disease activity and the pathological response of mice with chronic colitis (Fig. 5). In contrast to mice with acute colitis, the expression of IFN-γ mRNA in the splenocytes of DSS-treated mice with chronic colitis decreased, whereas the expression of IL-17A expression in the splenocytes increased slightly. SJMHE1 treatment decreased the expression of IL-17A mRNA and increased the expression of IL-10 mRNA in the splenocytes and colon compared with the respective expression levels in the DSS- and/or DSS/PBS-treated mice with chronic colitis (Fig. 6). The proportion of Th1 cells in the splenocytes of the mice with DSS-induced chronic colitis was lower than that in mice with acute colitis. By contrast, the percentages of Th2 and Th17 cells in mice from the DSS-induced chronic colitis increased compared with those in the normal (control) mice (Fig. 7). However, SJMHE1 treatment reduced the percentage of Th17 cells and increased the proportion of Th1 and Treg cells in the splenocytes. SJMHE1 also increased the percentage of Th1 and Treg cells but slightly reduced the percentage of Th17 cells in MLNs (Fig. 8). These results suggest that SJMHE1 could inhibit Th2- and Th17-mediated chronic colitis in mice through the upregulation of Th1 and Treg cells. DSS-induced colitis was originally considered to be a Th1- or Th17-mediated inflammation [7, 39], and most of these findings are based on an acute colitis model, i.e. oral administration of DSS for 7 days [40]. However, chronic colitis can be induced by multiple cycles of DSS, with a histopathology similar to that of UC, and UC is a Th2-dominated inflammation [41]. Dieleman and colleagues [42] demonstrated that DSS-induced chronic colitis is characterized by Th1 and Th2 cytokines. Th17 cells have been reported to participate in the development of DSS-induced chronic colitis in mice [43, 44]. Interestingly, SJMHE1 inhibits not only Th1- and Th17-mediated acute colitis but also Th2- and Th17-mediated chronic colitis in mice. A similar helminth molecule is ES-62 from *A. vitae*. ES-62 inhibits inflammation by restoring the immune balance regardless of inflammatory phenotype [44–46]. As a small molecule peptide from helminths that co-evolved with humans, SJMHE1 might be a potential drug candidate for the treatment of various inflammatory diseases, such as asthma, arthritis, and colitis.

## Conclusions

In conclusion, SJMHE1 from *S. japonicum* alleviates DDS-induced acute and chronic colitis in mice. SJMHE1 treatment regulates cytokine expression and Th balance in the splenocytes and inflammatory site (colon or MLNs) from mice with acute and chronic colitis. SJMHE1 may be a novel therapeutic agent that can be exploited for the treatment of IBD without immunogenicity concerns.

## Data Availability

The dataset supporting the conclusions of this article is included within the article.

## Abbreviations

CD: Crohn’s disease
DAI: Disease Activity Index
DSS: Dextran sulphate sodium
ES: Excretory/secretory
H&E: Hematoxylin and eosin
IBDs: Inflammatory bowel diseases
IFA: Incomplete Freund’s adjuvant
MLNs: Mesenteric lymph nodes
PC: Phosphorylcholine
Th: Helper T (cell)
TNBS: Trinitrobenzenesulfonic acid
Treg: Regulatory T (cell)
TSO: *Trichuris suis* ova
UC: Ulcerative colitis
